# SPECT/CT imaging in bone scintigraphy of a case of clavicular osteoma

**Published:** 2014

**Authors:** Yuka Yamamoto, Yoshihiro Nishiyama

**Affiliations:** 1 Department of Radiology, Faculty of Medicine, Kagawa University, Kagawa, Japan

**Keywords:** Bone scintigraphy, Osteoma, SPECT/CT, Tc-99m HMDP

## Abstract

Osteoma is a benign bone-forming tumor that usually arises in the craniofacial bones and rarely in the long bones. Clavicular involvement is extremely rare. We report a 51-year-old woman with osteoma of the left clavicle. Radiograph of the left shoulder showed a well-defined lobulated blastic mass in the proximal and mid-portion of the left clavicle. Bone scintigraphy was performed 4 hours after an intravenous injection of Tc-99m hydroxymethylene diphosphonate (HMDP). Whole-body image showed a focus of intensely increased uptake in the clavicle. Single photon emission computed tomography / computed tomography (SPECT/CT) images were also acquired and clearly showed intense uptake at the tumor site. Integrated SPECT/CT imaging supplies both functional and anatomic information about bone the SPECT imaging improves sensitivity compared with planar imaging, the CT imaging provides precise localization of the abnormal uptake, and information on the shape and structure of the abnormalities improves the specificity of the diagnosis.

## Introduction

Osteoma is a benign bone-forming tumor and clavicular involvement is extremely rare. We report a case of clavicular osteoma undergoing bone scintigraphy who demonstrated intense uptake in the clavicle, for which further single photon emission computed tomography / computed tomography (SPECT/CT) imaging was performed to characterize and localize the lesion.

## Case Report

**Figure 1:**
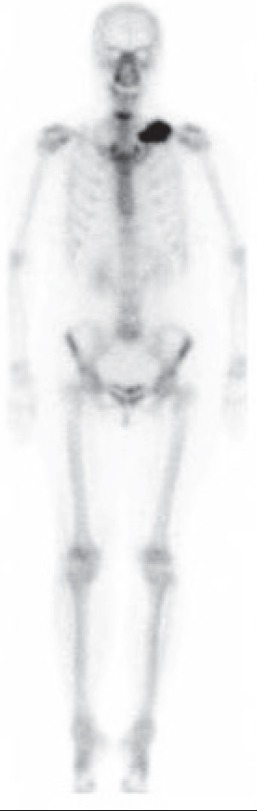
Whole-body anterior image shows a focus of intensely increased uptake in the proximal and mid-portion of the left clavicle

**Figure 2:**
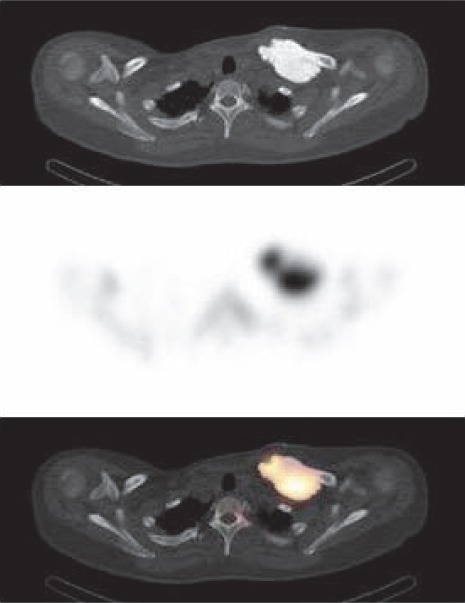
The transaxial CT image (upper section) identifies a dense lesion with well-defined contours in the left clavicle. There is intensely increased uptake on the corresponding SPECT image (middle section) and SPECT/CT fusion image (lower section)

The present case was a 51-year-old woman who presented with a left clavicular mass. The tumor had grown very slowly since the age of 21 years when the lesion was detected incidentally on plain chest X-ray. Radiograph of the left shoulder showed a 70×35 mm, well-defined lobulated blastic mass in the proximal and mid-portion of the left clavicle. Bone scintigraphy was performed 4 hours after an intravenous injection of Tc-99m hydroxymethylene diphosphonate (HMDP). Whole-body image showed a focus of intensely increased uptake in the clavicle (Figure 1). SPECT/CT images were also acquired and provided precise localization of the abnormal uptake (Figure 2). Histological findings were consistent with an osteoma.

## Discussion

Osteoma is a benign ostogenic tumor that usually arises in the craniofacial bones and rarely in the long bones (1, 2). Clavicular involvement is extremely unusual (3, 4). The radiological appearance of osteoma is usually that of a round or ovoid, sharply marginated blastic mass with no associated soft tissue mass (5). On bone scintigraphy, a significant focal increased uptake is noted, corresponding to the area of the blastic mass (6). The most important differential diagnosis is parosteal osteosarcoma. In comparison with parosteal osteosarcoma, osteoma usually presents as a homogeneous and dense lesion without an accompanying soft-tissue mass, cortical destruction, or intramedullary invasion (1). This is perhaps best appreciated on CT scans (1). Integrated SPECT/CT imaging supplies both functional and anatomic information about bone: the SPECT imaging improves sensitivity compared with planar imaging, the CT imaging provides precise localization of the abnormal uptake, and information on the shape and structure of the abnormalities improves the specificity of the diagnosis (7).
